# Low Level of *Her-2* Locus Amplification by Fluorescent In Situ Hybridization Does Not Correlate with Her-2 Protein Overexpression by Immunohistochemistry in Barrett's Esophagus

**DOI:** 10.1155/2010/382582

**Published:** 2010-06-09

**Authors:** Agnieszka M. Rygiel, Francesca Milano, Fiebo J. ten Kate, Jacques J. G. H. M. Bergman, Kausillia K. Krishnadath

**Affiliations:** ^1^Department of Gastroenterology and Hepatology, Academic Medical Center, Meibergdreef 9, 1105 AZ Amsterdam, The Netherlands; ^2^Center for Experimental and Molecular Medicine, Academic Medical Center, Meibergdreef 9, 1105 AZ Amsterdam, The Netherlands; ^3^Department of Pathology, Academic Medical Center, Meibergdreef 9, 1105 AZ Amsterdam, The Netherlands

## Abstract

An accurate evaluation of the *Her-2* status has important prognostic and therapeutic implications in many carcinomas. The aim of the study was to correlate *Her-2* locus (17q11.2) amplification and chromosome 17 gains as assessed by fluorescent in situ hybridization (FISH) with Her-2 protein overexpression by immunohistochemistry (IHC) in patients with Barrett's esophagus (BE) and esophageal adenocarcinoma (EAC). We analyzed 34 patients with *Her-2* amplification and/or chromosome 17gains using FISH on brush cytology specimens. Seven patients (21%) showed high *Her-2* locus amplification (*Her-2*: Cep17 ≥ 5 : 1), 5 (15%) showed low *Her-2* locus amplification (*Her-2*: Cep17 ≥ 2 < 5 : 1), and 22 (65%) displayed gains of chromosome 17 only. Further, we confirmed *Her-2* amplification on corresponding biopsies that were taken at the same occasion as the cytologybrushings. Then, we compared the FISH results with IHC data obtained from the corresponding biopsies and showed that low level of Her-2 amplification does not correlate with Her-2 protein overexpression (score +3/+2; *P* = 1), in contrast to the high amplification level (*P* = .001). Thus, in our population of BE and EAC patients, low level of *Her-2* amplification does not result in detectable level of Her-2 protein as assessed by IHC.

## 1. Introduction

Barrett's esophagus (BE) is a premalignant condition of the distal esophagus that is associated with an increased risk of developing esophageal adenocarcinoma (EAC) [1, 2]. In recent years, the incidence of BE and EAC has been increasing dramatically and death from EAC has become a major problem [1, 3]. Since long-term survival of EAC patients is highly dependent on early diagnosis, detection of BE patients at high risk for malignant progression has become crucial [4]. The present endoscopic and histopathologic surveillance of BE patients have been proven to be insufficient for effective identification of high-risk patients [5–7]. Evaluation of objective, molecular markers may lead to a better rationale for surveillance programs, as well as, targeted therapeutic strategies.

*Her-2(neu) *is a proto-oncogene localized on chromosome 17q, which encodes for a transmembrane tyrosine kinase growth factor receptor (Her-2/c-erB-2) [8]. Amplification of the *Her-2* gene and Her-2 protein overexpression has been studied in many malignancies, but most extensively in breast cancer and its precursor lesions [9]. In breast cancer, Her-2 overexpression has been correlated with poor prognosis or a lack of response to chemotherapy [10, 11]. However, with the antibody-based (Tastuzumab/Herceptin) therapeutic approach as an adjunctive treatment for *Her-2* positive breast cancer patients, the prognosis of this patient group has significantly improved [12, 13]. 

*Her-2* amplification and its protein overexpression have also been reported in dysplasia and EAC associated with BE. Several immunohistochemical studies on BE, suggest that Her-2 protein overexpression is a frequent and early event [14, 15], whereas others indicate that it is much less common and occurs late during the process of BE progression into EAC [16, 17]. Most of the studies that investigated *Her-2* gene amplification describe this as a rather late event in BE progression, which is present only in HGD and EAC cases [18, 19], while some indicate that *Her-2* amplification can already be seen in LGD [20]. Thus, the association of *Her-2* gene amplification and Her-2 protein overexpression during the process of BE progression is still unclear. Moreover, there seems to be an important discrepancy between results from immunohistochemistry (IHC) and gene amplification studies for determining Her-2 status. An accurate detection of the Her-2 status may help to identify high-risk subpopulations in BE surveillance cohorts, and to select for those EAC patients who may benefit from Her-2-targeted therapies [21]. 

The most widely used assays to determine *Her-2* status is immunohistochemistry (IHC) for the detection of protein overexpression, and DNA fluorescent in situ hybridization (FISH) for assessing the locus copy number. Although, IHC staining is the predominant method utilized, it can be significantly affected by technical issues, that is, tissue fixation, specificity of the antibody, and variation in quantification and interpretation of the intensity of the staining [22, 23]. DNA-FISH is not as widely available as IHC since it requires appropriate equipment and optimization for each tissue type, but, it is quantitatively accurate and highly reproducible [24]. This technique can be successfully applied on both archival paraffin biopsies [19, 20, 25] and on brush cytology samples [26, 27]. The important advantages of applying brush cytology to BE patients includes simplicity, lower cost, and the potential to sample a larger area of the BE epithelium when compared to taking random biopsies. Cytology samples are also more reliable for the enumeration of FISH signals, since there is no artifact caused by nuclear truncation as is the case when using tissue sections [28]. 

Recently, we have evaluated the frequency of *Her-2* locus and chromosome 17 abnormalities using DNA-FISH on brush cytology specimens of BE patients with different stages of dysplasia [27]. The aim of the present study was to compare the *Her-2* locus amplification and/or chromosome 17 gains as determined by DNA-FISH on BE/EAC brush cytology specimens to Her-2 protein overexpression as assessed by IHC on corresponding biopsies that were taken at the same time as the brush cytology specimens.

## 2. Materials and Methods

### 2.1. Patients

A total of 34 of BE patients showing *Her-2*/chromosome 17 abnormalities were included in this study. Out of these 34 cases, 22 patients showed gain of chromosome 17, and 12 patients had gain of chromosome 17 and/or *Her-2* locus amplification. The cases were either retrieved from our previous DNA-FISH surveillance study on brush cytology specimens [27] or from an ongoing study in which EAC patients are screened for *Her-2* amplification status by DNA-FISH. The patients underwent endoscopy at the Academic Medical Center in Amsterdam between 2002 and 2009. During endoscopy, brush cytology specimens and corresponding biopsies were taken for FISH, IHC, and histopathological analysis. In case of BE, the biopsies were taken at least per 2 cm in 4 quadrants and of all suspected visible lesions using the protocol of Reid et al. [29]. In the EAC cases, biopsies were taken from the mass, and if present also from the adjacent Barrett's mucosa. All BE patients were on long-term proton pump inhibition of 40 to 80 mg daily to prevent reflux esophagitis. The Ethics Committee of the Academic Medical Center approved the study. All patients signed informed consent for the use of their biopsy and brush cytology material.

### 2.2. Brush Cytology

Cytological brush material was sampled using the Wilson-Cook (Winston-Salem, NC) brush type LCB-220-3-1.5-S as described previously [27]. From the cell suspension obtained from brushing a single layer of the cells on a glass slide was generated using the Cytospin (Shandon Cytospin 4 Cytocentrifuge, Thermo, Waltham, MA). The cytospin procedure was performed as described previously [27]. After the procedure the cytospin slides were dried at RT, and then stored at −80°C.

### 2.3. Fluorescent In Situ Hybridization (FISH) on Brush Cytology and Tissue Samples

Dual color probe was used combining chromosomal centromeric probe (CEP) for chromosome 17 SpectrumGreen with the locus specific probe (LSI) for *Her-2* (17q11.2-q12) SpectrumOrange (Vysis, Downers Grove, IL). DNA-FISH on brush cytology was performed as described previously [27]. Additionally, DNA tissue FISH was preformed in the subset of the patients, to determine correlation between abnormalities as assessed by FISH on brush cytology and tissue samples [30].

### 2.4. Scoring of FISH Signals

As described previously, after the FISH procedure, 100 to 200 interphase nuclei of BE cells were scored per slide by an experienced scorer (A. M. Rygiel) using Olympus BX61 fluorescent microscope (Germany) [27]. The cases were evaluated without prior knowledge of histology findings. Damaged cells and cells with indistinct and blurry signals were excluded from the analysis. The categories of *Her-2* locus abnormalities were determined by calculating the ratio of *Her-2* locus signals (red) to CEP17 signals (green) as described previously [31]. The following categories were distinguished: A ratio <2 were considered as having no amplification, ratio's ≥2 and <5 were considered as a low amplification, and ratio ≥5 was considered as a high amplification. More then two green signals (CEP 17) accompanied by the same number of red signals (*Her-2* locus) was considered to be indicative of gain of chromosome 17 (ratio 1 : 1). Following these criteria the cases were classified as displaying a gain of chromosome 17 and a low-or high-level amplification of *Her-2 *locus (cutoff ≥3% of abnormal nuclei). The cutoff value was obtained from counts in the normal squamous epithelium taken from 20 BE patients without dysplasia and calculated as the mean percentage of squamous nuclei with signal gain plus 3xSD (standard deviation) as described previously [27].

### 2.5. Immunohistochemistry (IHC)

 Immunohistochemistry (IHC) was performed on archival material from paraffin embedded tissue obtained during the same endoscopy procedure as the brush cytology. IHC for Her-2 protein (antibodies/Her-2/neu/c-erbB-2 Ab-17 clone e2-4001+ 3 B5, mouse monoclonal, Neomarkers, Stratech Scientific Ltd, Cambridgeshire, UK) was performed according to a standard IHC protocol. Briefly, paraffin sections were deparaffinised and rehydrated in graded alcohols. Endogenous peroxidase activity was quenched with 0.3% hydrogen peroxide in methanol for 20 minutes and then washed (3 × 5 minutes in PBS). Antigen retrieval was performed by boiling slides for 10 minutes in 0.01 M Sodium Citrate Ph 6.0. Nonspecific binding sites were blocked with 5% goat serum in PBS for 10 minutes, and then washed (3 × 5 minutes in PBS). Slides were incubated with the primary antibodies diluted 1 : 2000 in Normal Antibody Diluent (Scytek, Logan, Utah, USA) for 60 minutes. After washing (3 × 5 minutes in PBS), postantibody blocking solution (Immunologic, Duiven, The Netherlands) diluted 1 : 2 in PBS was applied for 15 minutes. After washing (3 × 5 minutes in PBS), slides were then incubated with biotinylated secondary antibodies diluted 1 : 2 in PBS (Poly-HRP-Goat anti Mouse IgG, Immunologic) at room temperature for 30 minutes. Slides were washed (3 × 5 minutes in PBS), and then the peroxidise activity was detected with “Fast DAB” (3,3′-diaminobenzidine, Sigma, St Louis, MO) with 0.05% hydrogen peroxide. Finally, sections were counterstained with Mayer's haematoxylin, dehydrated and mounted with Pertex under cover slips. Her-2 protein expression was evaluated by an experienced pathologist (F. J. ten Kate) according to the scoring system recommended by the DACO HercepTest. No staining at all or membrane staining in <10% of the epithelial cells was considered negative (score 0). Faint or barely perceptible, incomplete membrane staining in >10% of the epithelial cell was scored +1. Weak to moderate staining of the entire membrane in >10% of the epithelial cells was scored +2, and strong staining of the entire membrane in >10% of the epithelial cells resulted in a score +3.

### 2.6. Statistical Analysis

Differences in frequencies were tested using a Fisher's exact test (2-sided) and statistical significance was set at a *P*-value of <.05. The statistical analyses were conducted using SPSS software (version 12.0; SPSS, Inc, Chicago, IL).

## 3. Results

### 3.1. Patients

Of the 34 cases included in this study, 31 were male and 3 female with a median age of 60 (range 26–84). In the BE cases, the median BE length was 6 cm (range 1–13 cm). These BE cases included 4 patients with ND, 5 patients with IND or LGD, and 13 patients with HGD. There were 12 patients with EAC. Nine out of the 12 EAC patients were staged according to the Union International Control Center TNM system. The EAC patients included 5 cases with T1/T2N0M0, 2 cases with T3N0M0, and 2 cases with T3N1M0 stage.

### 3.2. Chromosome 17 and Her-2 Locus Copy Number as Assessed by FISH on Brush Cytology Samples

Twenty two patients (4 ND, 5 IND/LGD, 4 HGD, and 9 EAC) displayed gains of chromosome 17 and 12 patients (9 HGD and 3 EAC) showed *Her-2* locus amplification. Of the 12 cases with the *Her-2 *locus amplification 5 patients (41%) displayed a low level, and 7 patients (58%) a high level of the *Her-2* locus amplification.

### 3.3. Confirmation of the FISH Results as Found in the Brush Cytology Specimens on Corresponding BE Biopsy Samples

In seven cases with *Her-2* locus amplification detected in the cytology samples, FISH was also performed on biopsies that were taken at the same occasion as the cytology brushings. Three cases showed a low level of *Her-2* locus amplification and 4 cases displayed a high level of the amplification. In all seven cases the amplifications as found in the cytology specimens were also seen in the corresponding biopsy samples (Table 1).

### 3.4. Correlation between Chromosome 17 and Her-2 Locus Copy Numbers by FISH on Brush Cytology and Her-2 Overexpression as Determined by IHC on Biopsies

The comparison between chromosome 17 status, the levels of *Her-2* locus amplification, and Her-2 protein overexpression is presented in Tables 1 and 2. Of the 7 cases with a high level of *Her-2* amplification, 5 (72%) showed strong overexpression of the protein (+3), and two cases (28%) showed moderate or faint Her-2 overexpression (+1/+2). Of the 5 patients displaying a low level of *Her-2* amplification, only one case (20%) showed moderate Her-2 overexpression (+2) while the rest were negative for protein overexpression. Of the 22 cases with gain of chromosome 17, 3 cases (14%) showed moderate Her-2 overexpression (+2), whereas the remaining cases showed faint or no staining of the Her-2 protein (0/+1). 

Statistical analysis showed that there is a significant difference between low levels and high levels of *Her-2* locus amplification with respect to Her-2 protein overexpression as detected by IHC (*P* = .028). When we compared *Her-2* amplified cases to cases with no amplification, we found that only a high level of the amplification correlates with strong or moderate overexpression of the Her-2 protein (*P* = .001). In contrast, there is no correlation between low amplification of the *Her-2* locus and Her-2 protein overexpression (*P* = 1; Table 2).

## 4. Discussion

It is generally believed that *Her-2* locus amplification is always coupled with strong *Her-2* protein overexpression, while gains of chromosome 17 usually do not result in Her-2 protein overexpression [19, 20, 32, 33]. For most cancers, daily practice is to use IHC to determine Her-2 overexpression, and in case of positive IHC staining, FISH is applied to confirm gene amplification. This is generally done to rule out false positive staining by IHC. In BE and EAC it is, however, not clear whether this set up would be suitable to identify all patients with abnormal *Her-2* gene status. Therefore, in this study, we were interested in the miss rate (false negativity) of IHC to detect cases with *Her-2* locus amplifications as assessed by FISH. Hereto, we correlated *Her-2* locus amplification and/or chromosome 17 gains with Her-2 protein overexpression in BE and EAC cases. We found important differences between the level of *Her-2* locus amplification and Her-2 protein overexpression. We demonstrated that low levels of *Her-2* amplification (ratio of *Her-2*: Cep17 ≥ 2 < 5 : 1) do not correlate with IHC (*P* = 1), in contrast to high amplification levels (ratio of *Her-2*: Cep17 ≥ 5 : 1), which shows a significant correlation with Her-2 protein overexpression (*P* = .001).

The majority of our cases (5/7, 72%) with a high level of the *Her-2* locus amplification in the brush cytology specimen showed strong protein overexpression (+3 score) in the biopsy specimen, which is in agreement with literature data showing a high correlation between IHC +3 staining and amplifications detected by FISH [31, 34]. However, two of our HGD cases with high level of *Her-2* amplification showed faint or moderate Her-2 overexpression. This discordance may be due to heterogeneity of the lesion or to the subjective interpretation of the staining intensity [23]. 

More importantly, we demonstrated that a low level of *Her-2* locus amplification did not result in strong (+3) Her-2 overexpression, while moderate (+2) Her-2 staining was seen in only one case. One could argue that this finding may be due to our methodology, since we applied DNA-FISH on brush cytology samples and compared these results to IHC on biopsy specimens. Theoretically, in case of tumor heterogeneity, random biopsies may have missed areas with the *Her-2 *locus amplification due to sampling errors, while brush cytology in principal samples the whole or the majority of the BE surface and may give a better representation of the different cellular clones that may coexist in BE [35]. To investigate whether the discrepancy between the *Her-2* locus amplification in the cytology specimens and IHC on biopsies was due to sampling errors when taking biopsies, we also performed FISH on the biopsies in several cases. In all these cases, we were able to confirm the *Her-2* locus amplification in the corresponding biopsy sample. Therefore, our results are very unlikely to be due to a bias caused by biopsy sampling errors. Besides, our finding is in agreement with literature data. A similar discordance between low levels of* Her-2* amplification and protein overexpression was also found in ovarian tumors comparing IHC with FISH on biopsy samples [34]. Moreover, studies on breast cancer compared IHC with FISH, and demonstrated that only a minority of cases (3%–7%) with low levels of *Her-2* amplification show protein overexpression [36–38]. This indicates that in general DNA-FISH seems to be a more sensitive technique then IHC to detect low levels of *Her-2* gene status changes. As expected, we found that the majority of our cases (86%) with gains of chromosome 17 only, showed no Her-2 protein overexpression, while three of these cases (14%) showed moderate overexpression. These observations are consistent with studies evaluating Her-2 status by IHC and FISH on BE/EAC tissue sections, showing moderate (+2) Her-2 protein overexpression in some cases but no association with strong (+3) overexpression [19, 20]. Thus, our results actually indicate that the levels of Her-2 protein as a result of low *Her-2* locus amplification and gains of chromosome 17 are in general too low for detection by IHC, while FISH accurately can detect these cytogenetic abnormalities. This is probably because FISH, as demonstrated in breast cancer studies, is quantitatively accurate and very reproducible [24]. Another explanation may be that particularly in cases with low level of *Her-2* status, gene transcription and posttranscriptional or posttranslational events could be down-regulated or abnormal, ultimately leading to low Her-2 protein levels or abnormal epitope production. Alternatively, tissue preservation could have been insufficient, leading to protein degradation resulting in faint or negative staining. 

So far, there is no consensus with regard to the optimal test for Her-2 assessment. Although, IHC staining is the predominant method utilized, it can be significantly affected by technical issues, especially in archival fixed paraffin tissues, resulting frequently in false positive results since the scores are based on staining intensity [39]. Therefore, in breast cancers it is strongly recommended that IHC +2 cases are confirmed by FISH for a more appropriate selection of candidate patients for targeted therapy, for instance with the anti Her-2 monoclonal antibody Trastuzumab [36]. In our set up, however, we were able to show that IHC gives false negative results mostly in cases with low level of *Her-2* amplification. In this respect, it is important to realize that low levels of *Her-2* amplification in breast cancer is regarded as an indication for immunotherapy with Trastuzumab [40]. Moreover, it is expected that in the upcoming era BE patients with Her-2 positive esophageal adenocarcinomas will also benefit from this therapy and the first phase I/II trials have been already published [21, 41].

In summary, we showed that DNA-FISH on brush cytology samples is a representative and useful diagnostic tool, which at least in cases with low level of the *Her-2 *locus amplification, is superior to IHC on biopsy. Although more studies with larger sample size need to be performed to confirm our findings, we suggest that FISH should be the first method of choice for accurate detection of *Her-2* status in BE and EAC patients. This is of importance since an accurate assessment of *Her-2* status in BE associated EAC and other malignancies is highly relevant for proper selection of patients that are eligible for treatment with Trastuzumab or other Her-2 targeted molecular strategies.

## Figures and Tables

**Figure 1:**
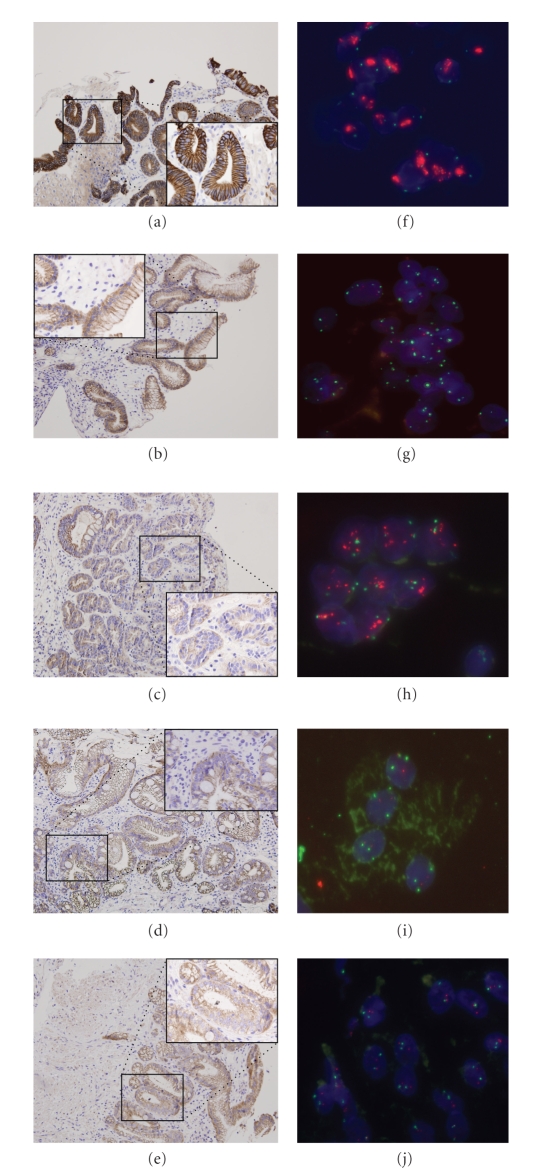
Her-2 protein and *Her-2* locus status as determined by IHC on BE biopsy and FISH on BE brush cytology specimens. (a) Strong overexpression of Her-2 protein (+3) in HGD, (b) moderate overexpression of Her-2 protein (+2) in EAC, (c) no overexpression of Her-2 protein (score 0) in EAC, (d) no overexpression of Her-2 protein (score 0) in LGD, (e) no overexpression of Her-2 protein (score 0) in ND, (f) high level of *Her-2* locus amplification (clusters) in a cytology sample of a HGD case—CEP 17 (green) and *Her-2* locus (red), (g) gain of chromosome 17 in a cytology sample of an EAC case—CEP 17 (green) and *Her-2* locus (red), (h) low level of *Her-2* amplification (ratio ≥2 < 5  :  1 of *Her-2*: Cep17) in a cytology sample of an EAC case, CEP 17 (green) and *Her-2* locus (red), (i) gain of chromosome 17 in a cytology sample of a LGD case—CEP 17 (green) and *Her-2* locus (red), and (j) two copies of chromosome 17 (disomy) and Her-2 locus (normal pattern) in a cytology sample of a ND case—CEP 17 (green) and *Her-2* locus (red).

**Table 1:** Her-2 protein overexpression and *Her-2* locus/chromosome 17 status in BE and EAC cases.

No.	Histology	IHC	Brush cytology FISH		Tissue FISH	
		Her-2 expression	*Her-2 *locus amplification	CEP17	*Her-2 *locus amplification	CEP17
91	ND	0	—	Gain	Nd	Nd
237	ND	0	—	Gain	Nd	Nd
75	ND	0	—	Gain	Nd	Nd
148	ND	0	—	Gain	Nd	Nd
59	IND	1	—	Gain	Nd	Nd
134	IND	0	—	Gain	Nd	Nd
273	IND	1	—	Gain	Nd	Nd
98	LGD	0	—	Gain	Nd	Nd
255	LGD	0	—	Gain	Nd	Nd
3	HGD	0	—	Gain	Nd	Nd
4	HGD	3	High (50)	Gain (42)	High (39)	Gain (13)
5	HGD	0	Low (5)	Gain (71)	Low (10)	Gain (59)
152	HGD	0	—	Gain	Nd	Nd
167	HGD	1	High (8)	Gain (13)	Nd	Nd
170	HGD	2	Low (11)	—	Nd	Nd
173	HGD	3	High (50)	Gain (23)	Nd	Nd
193	HGD	0	Low (90)	Gain (6)	Low (55)	—
202	HGD	3	High (75)	Gain (83)	High (79)	Gain (84)
211	HGD	2	High (6)	Gain (11)	High (34)	Gain (34)
235	HGD	0	—	Gain	Nd	Nd
236	HGD	1	Low (20)	Gain (13)	Low (10)	Gain (53)
247	HGD	2	—	Gain	Nd	Nd
264	EAC	1	—	Gain	Nd	Nd
265	EAC	2	—	Gain	Nd	Nd
270	EAC	0	—	Gain	Nd	Nd
223	EAC	0	Low (8)	Gain	Nd	Nd
232	EAC	1	—	Gain	Nd	Nd
233	EAC	2	—	Gain	Nd	Nd
251	EAC	1	—	Gain	Nd	Nd
254	EAC	0	—	Gain	Nd	Nd
200	EAC	3	High (82)	—	Nd	Nd
274	EAC	0	—	Gain	Nd	Nd
276	EAC	3	High (50)	Gain (63)	High (42)	Gain (42)
250	EAC	0	—	Gain	Nd	Nd

FISH—fluorescent in situ hybridization; (—) indicates absence of certain abnormality; (gain)—ratio 1 : 1 of *Her-2*: Cep17, the number of signals in different cases varied from 3 to 6 signals per cell; (low)—low level amplification—ratio ≥2 < 5 : 1 of *Her-2*: Cep17; (high)—high level amplification—ratio ≥5 : 1 of *Her-2*: Cep17; in the brackets % of abnormal cells

IHC—immunohistochemistry; IHC score 0—no staining, IHC score +1—faint staining, IHC score +2—moderate staining, IHC score +3—strong staining;

ND—no dysplasia; IND—indefinite for dysplasia; LGD—low grade dysplasia; HGD—high grade dysplasia.

**Table 2:** Frequencies of Her-2 protein overexpression in BE/EAC patients with respect to high and low levels of *Her-2* locus amplification versus no amplification (Cep17 gain).

FISH	IHC		P*
	0/+1	+2/+3	
	No./total No. (%)		
No amplification (Cep 17 gain)	19/22 (86)	3/22 (14)	.001
*Her-2* high amplification	1/7 (14)	6/7 (86)	
No amplification (Cep17 gain)	19/22 (86)	3/22 (14)	1
*Her-2* low amplification	4/5 (80)	1/5 (20)	

FISH—fluorescent in situ hybridization, IHC—immunohistochemistry, *Fisher exact test.

## Abbreviations

ND: no dysplasia
LGD: low grade dysplasia
HGD: high grade dysplasia
EAC: esophageal adenocarcinoma
FISH: fluorescent in situ hybridization
IHC: immunohistochemistry.
